# Why is the *New York Times* Writing so Much about Alzheimer's Disease Therapies?

**DOI:** 10.1100/tsw.2010.185

**Published:** 2010-09-14

**Authors:** Tamas Bartfai

**Affiliations:** Molecular and Integrative Neurosciences Department, The Scripps Research Institute, La Jolla, CA, USA

**Keywords:** New York Times, Alzheimer’s disease, familial AD, amyloidosis, amyloid beta-peptide, memory, cognition, memantine, Gleevec, β-secretase, γ-secretase, preventive medicine, prevention trials

## Abstract

Can we go back to proving that drugs work in preventing, postponing, and ameliorating familial Alzheimer's disease (ad)? Ad is so devastating that there is a great public interest in the drug discovery process as evinced by the sheer number of articles in the serious popular press. The presently available, yet poorly performing, drugs have been approved despite their multiple peripheral side effects. The research on disease-modifying agents for the treatment of ad is largely focused on reducing amyloid plaques. However, it is now clear that companies and researchers alike are losing hope in finding an efficacious therapy rapidly that works in ad patients who are already cognitively impaired, and that people who staked their scientific and professional career on finding a cure for ad based on the amyloid hypothesis are shaken by the series of failed clinical trials within a short period of time. It is emerging that we may start to treat ad far too late to be able to make any significant slowing of the disease or postponing the onset of the symptoms of the disease. The history of drug development for other diseases should encourage us to focus on patients in whom we can identify the genetic markers associated with familial ad. Then when we have an efficacious and very safe drug, we will be able to establish its efficacy on, most importantly, cognition, but also at the level of plaques. This will provide the pharmacological evidence needed to show that it is worth fighting amyloidosis because it saves memory. We have a successful and lucrative history of preventive medicine on a large scale, all we need now is the foresight and will to switch strategy and no longer look for a magic bullet to fix ad, but to discover drugs that will delay and prevent the onset of ad, drugs that may be safely taken by symptom-free patients who are vulnerable and susceptible to ad. The initial population that might be preventatively treated against ad would indeed be those with genetic predisposition. While prevention trials are long and expensive as emphasized by the industry, the market for a safe and effective drug would be extended to the large number of patients susceptible to sporadic ad. Since the highest risk factor for sporadic ad is age, this would be an extraordinarily large market.

Can we go back to proving that drugs work in preventing, postponing, and ameliorating familial Alzheimer's disease (AD)?

The research on disease-modifying agents for the treatment of AD, which affects one in ten people over the age of 70 and almost half of the population over 85, is largely focused on reducing amyloid plaques built from the aggregates of amyloid beta peptide (Aβ) 1-40/42 and numerous other proteins that are found in neuropathological examination of victims of familial AD and sporadic AD, although amyloid load does not necessarily equal AD. Presently approved drugs include acetylcholine esterase inhibitors and an NMDA receptor antagonist, memantine, for treatment of cognitive decline in mild and moderate AD as diagnosed by neuropsychological test batteries. The trials leading to approval of these drugs were 12–18 months long, since in mild AD, the patients' cognitive decline is slow and to show that the drug slows this decline significantly, one needs both large groups of patients and long treatment time. The cognitive performance of patients treated with drugs vs. placebo diverged statistically significantly and, thus, these drugs were approved despite their multiple peripheral side effects.

To prevent formation of amyloid peptides and the plaques that, according to the amyloid hypothesis of AD, cause the disease symptoms, research aimed to develop inhibitors of the two endopeptidases, β- and γ-secretase, which together cleave the amyloid precursor protein (APP), giving rise to the amyloidogenic β-peptide 1-40/42, with aggregating and neurotoxic properties in its monomeric and oligomeric forms. Antibodies against this peptide as antigen were also developed, and it was shown that they can disaggregate plaques in transgenic animal brains and "pull out" amyloid peptides from the brain, something first believed to be impossible. The approaches targeting Aβ 1-40/42 by vaccination ran into serious problems when the vaccine caused life-threatening brain inflammation in a large number of patients in 2002 (but permitted in the autopsy material a clear view that the anti-Aβ antibodies had entered the brain and attached themselves to plaques). Lately, other anti-Aβ antibodies, made as recombinant proteins and to be used in a passive immunization approach, have been advanced to phase 2 clinical trials, in which, using a novel imaging agent, they have been shown to reduce plaque load, but also cause vascular swelling, in itself an alarming phenomenon. The significance with regard to safety of this swelling is yet to be understood[1,2,3].

The β-secretase inhibitors so far tested have failed to meet safety requirements and to improve cognitive performance, but several new molecules are in phase 1 trials. The γ-secretase inhibitor tested last month made cognitive performance worse. However, cognitive performance is the only thing patients and regulatory agencies should care about as no one has sleepless nights over having or not having plaques as long as memory serves us. It is apparent that the approved drugs, as well as the drugs being tested, have multiple side effects. The amyloid researchers, who dominate AD research and drug development, became alarmed and the opponents became loud. All this resurgent debate about the amyloid hypothesis of AD is not only in scientific journals, but also in such publications as the *New York Times* (NYT). Surely the NYT deals with major health care issues as a significant daily should, and it has an aging, well-educated readership that can and wants to understand the state of the art with respect to the treatment of AD (see, for example, recent articles [4,5,6,7,8,9,10,11]). No one can deny that the increase in AD patients as our society ages puts an ever-increasing burden on health care systems, for instance, when demanding three persons to cover an AD patient over every 24-h period, and in economical terms, thus motivating the NYT to deal with the issue of where we are with the much-awaited new therapies. The new therapies would also be multibillion dollar drugs. The present AD treatments are fairly low-efficacy/high-side-effect drugs that are given only to mild to moderate AD patients, but once started, they are prescribed for the rest of their lives. Even economy journalists following the fortunes of pharmaceutical companies have a stake in watching this development in a city like New York and in a paper like the NYT, as they predicted that the Wyeth-Pfizer Aβ antibody tested in passive immunization would surpass Lipitor's sales of $13 billion/year.

But, the real surprise is that it becomes clear that companies and researchers alike are losing hope in finding an efficacious therapy rapidly, and that people who staked their scientific and professional career on finding a cure for AD based on the amyloid hypothesis are shaken by the series of failed clinical trials within a short period of time. Although the amyloid hypothesis has a very solid scientific grounding, as the familial AD cases (Swedish, Dutch AD, etc. with early onset of AD) have all been shown to possess mutations in the APP or in a protein associated with it, presenilin, with the accumulating failures of expensive and large (and not always scientifically well-founded) clinical trials for AD, and the testing of an old antihistamine (like the recently phase-3 failed Dimebon[11]), pharmaceutical companies are also increasingly helplessly casting around for drug candidates, and the direction and these failures cannot be kept from the economy and science pages.

The NYT then finds a sunshine story[10]. Paul Greengard, the scientifically and personally appealing Nobel laureate in Physiology and Medicine 2000, has identified a new handle on γ-secretase, and this he achieved using a highly successful cancer drug, Gleevec, which unfortunately cannot be used as is to treat AD, but it may lead the way[12].

However, in all of these hopes-and-failures reports of new clinical trials and in scientific correspondence on the pages of the NYT, we do not discuss the emerging understanding that we may start to treat AD too late to be able to make any significant slowing of the disease or postponing the onset of the symptoms of the disease. It may not be that there is anything wrong with the amyloid hypothesis, but we are treating AD patients too late when the Aβ peptide in its different oligomeric and aggregate forms has already killed too many neurons. Although we have some minimal ability to generate new neurons (neuronal stem cells can divide and differentiate, but not everywhere, and they are far fewer than your reading about them would suggest), we are nowhere close to replacing the lost neurons in AD with new ones in the right place in the network to restore memory function.

In this situation, we have to look at the history of drug development for other diseases, where it has always been helpful to find a genetically based (familial) variant of the disease, because understanding the molecular mechanism of the pathophysiology and then treating patients in whom we can identify the genetic markers has provided the best clinical trials. Treatment was provided to those patients for whose mutations the drug was, in a focused manner, developed. While the trials are smaller, the response rates and the efficacy in the responders are often higher. Thus, if the drug is good and safe, we will seldom miss its efficacy and we understand its safety and dosing issues before trying it in large and more varied population. Moreover, there are many potential patients to recruit for these trials since there are many familial, genetically-based errors of amyloid metabolism, and we know how to diagnose them when the carriers are still symptom free with full cognitive ability. Also the amyloid-imaging techniques have made great strides in the past 2 years, although no one will take a drug that lowers amyloid plaque unless we can prove that this lowering of plaque load will be associated with less or no cognitive decline despite the genetic background of the familial AD patient.

So if the path is so clear, trying to treat a genetically well-defined group, why do we not try? Well, because the companies believe that they can access the large, sporadic AD market with millions of patients at once. They believe it will take too long (5 years) to prove that a drug propones or ameliorates the disease in familial AD carriers and because the drugs that affect the amyloidosis in the brain have poor side effect profiles, not making them attractive to take for 5 years, without proof that it will be of benefit to someone, who is asymptomatic, even if this patient knows that he or she has the genetic makeup predisposing him or her to early-onset AD.

Without diagnosis there is no treatment. It is unethical to inform a prospective patient that they will develop an incurable disease from which they cannot escape without also providing hope of helping them to avoid, postpone, or slow down the disease.

Yet we have very solid genetic foundations to select the familial AD patients (if we can indeed offer them anything). We have increasingly sophisticated imaging of amyloid plaques[13], and we have better and better neuropsychological tests to measure early signs of change and decline of cognitive performance. Thus when we have an efficacious and very safe drug, we will be able to establish its efficacy on, most importantly, cognition, but also at the level of plaques. This will provide the pharmacological evidence needed to show that it is worth fighting amyloidosis because it saves memory[14]. We have a successful and lucrative history of preventive medicine on a large scale, also first tested in familial cases (albeit in much shorter trials). The industry sells cholesterol-lowering statins for over $13 billion/year to people with high cholesterol. High cholesterol is not in itself a disease, but a wellproven risk factor for cardiovascular disease. People are willing to take expensive drugs for many years to prevent illness, but these drugs must have a better safety profile because they will be taken by people who are healthy. We have to spend energy and dollars on trials that convince these "prepatients" and the regulators that there is a benefit in taking these drugs. We have to implement the necessary long prevention trials. The National Institute of Aging and the Banner Institute are trying to do such prevention trials, but neither of these organizations has any record of having discovered or developed a safe and efficacious CNS drug. However, they will be able to tell us when industry has found one, as that is their mission.

The short cuts where we try to go into the seriously ravaged brain of the mild or moderate AD patient with the "magic bullet" simply do not work[6]. The development of AD drugs is too important and the scientific evidence for the amyloidosis involvement in some, and maybe in all, sporadic cases of AD is too strong to cast aside, especially when nothing replaces it. We cannot continue searching for the shortcuts, whether these be small molecules, or biologicals, whether they will regulate the secretases directly or indirectly, or the formed amyloid. They must also be so safe that we can convince asymptomatic people to take them for many years. The companies that will not give up, that will go for the long road instead of looking for the short cut, as they do now, will be richly rewarded as the market grows.

We are getting older and the highest risk factor for sporadic AD is age.
